# School-Based Health Centers, Access to Care, and Income-Based Disparities

**DOI:** 10.1001/jamanetworkopen.2023.34532

**Published:** 2023-09-18

**Authors:** Michel Boudreaux, Jun Chu, Brandy J. Lipton

**Affiliations:** 1 Department of Health Policy and Management, University of Maryland, College Park; 2 Department of Sociology, Anthropology, and Public Health, University of Maryland, Baltimore County, Baltimore; 3 Department of Health, Society, and Behavior, University of California, Irvine

## Abstract

**Importance:**

School-based health centers (SBHCs) are primary care clinics colocated at schools. SBHCs have the potential to improve health care access and reduce disparities, but there is limited rigorous evidence on their effectiveness at the national level.

**Objective:**

To determine whether county-level adoption of SBHCs was associated with access, utilization, and health among children from low-income families and to measure reductions in income-based disparities.

**Design, Setting, and Participants:**

This survey study used a difference-in-differences design and data from a nationally representative sample of children in the US merged with SBHC indicators from the National Census of School-Based Health Centers. The main sample included children aged 5 to 17 years with family incomes that were less than 200% of the federal poverty level observed in the National Health Interview Survey, collected between 1997 to 2018. The sample was restricted to children living in a county that adopted a center between 2003 and 2013 or that did not have a center at any time during the study period. Analyses of income-based disparities included children from higher income families (ie, 200% or higher than the federal poverty level). Data were analyzed between January 2020 and July 2023.

**Exposure:**

County-by-year SBHC adoption.

**Main Outcomes and Measures:**

Outcomes included access (usual source of care, insurance status, barriers), ambulatory care use (general physician, eye doctor, dental, mental health visits), and health (general health status, missed school days due to illness). *P* values were adjusted for multiple comparisons using the sharpened *q* value method.

**Results:**

This study included 12 624 unweighted children from low-income families and 24 631 unweighted children from higher income families. The weighted percentage of children in low-income families who resided in counties with SBHC adoption included 50.0% aged 5 to 10 years. The weighted percentages of the race and ethnicity of these children included 36.7% Hispanic children, 25.2% non-Hispanic Black children, and 30.6% non-Hispanic White children. The weighted percentages of children in the counties that never adopted SBHCs included 50.1% aged 5 to 10 years. The weighted percentages of the race and ethnicity of these children included 20.7% Hispanic children, 22.4% non-Hispanic Black children, and 52.9% non-Hispanic White children. SBHC adoption was associated with a 6.4 percentage point increase in dental visits (95% CI, 3.2-9.6 percentage points; *P* < .001), an 8.0 percentage point increase in having a usual source of care (95% CI, 4.5-11.5 percentage points; *P* < .001), and a 5.2 percentage point increase in insurance (95% CI, 1.2-9.2 percentage points; *P* = .03). No other statistically significant associations were found with other outcomes. SBHCs were associated with relative reductions in income-based disparities to dental visits by 76% (4.9 percentage points; 95% CI, 2.0-7.7 percentage points), to insured status by 63% (3.5 percentage points; 95% CI, 1.3-5.7 percentage points), and to having a usual source of care by 98% (7.2 percentage points; 95% CI, 5.4-9.1 percentage points).

**Conclusions and Relevance:**

In this survey study with difference-in-differences analysis of SBHC adoption, SBHCs were associated with access to care and reduced income-based disparities. These findings support additional SBHC expansion.

## Key Points

**Question:**

What is the association between school-based health centers and access, use, and health among children from low-income families?

**Findings:**

In this survey study with difference-in-differences analysis using nationally representative survey data, school-based health centers were associated with increased dental visits (6.4 percentage points), having a usual source of care (8.0 percentage points), and insurance coverage (5.2 percentage points), among children from low-income families. Centers reduced income-based disparities in these measures.

**Meaning:**

The findings of this study support the continued expansion of school-based health centers.

## Teaser

This survey study examines whether county-level adoption of school-based health centers are associated with access, use, and health among children from low-income families using data from a sample of US children aged 5 to 17 years from 1997 to 2018.

## Introduction

School-based health centers (SBHCs) are primary care clinics that are colocated with or closely linked to schools. As of the 2016 to 2017 school year, there were 2584 SBHCs available to 6.3 million students.^1^ SBHCs are typically located in disadvantaged communities.^2^ Their multidisciplinary teams provide preventive care, acute care, and chronic disease management. Two-thirds of SBHCs provide behavioral health in addition to primary care, and 41% provide additional services, such as oral, vision, or nutrition services.^1^

SBHCs are a promising model that could reduce barriers, particularly for children from low-income families. Their location at schools reduces transportation challenges, alleviates the need for parents to take time away from work, and reduces difficulties in identifying a clinician that accepts their insurance.^3,4^ SBHCs can proactively promote services that families are unaware of or underestimate the value of receiving.

A number of studies suggest that SBHCs improve outcomes. However, many consider a single center or use cross-sectional comparisons.^5,6,7,8^ It is plausible that SBHCs substitute for care that students would obtain in the community and that existing evidence reflects unaddressed confounding.

The goal of this study was to strengthen the evidence on the association of SBHCs and access to care, use, and health in the low-income population and to document the role that SBHCs play in addressing income-based disparities. We used a nationally representative sample of school-age children and quasi-experimental methods to reduce the risk of confounding.

## Methods

The institutional review board at the University of Maryland determined the study exempt from review. This survey study follows the Strengthening the Reporting of Observational Studies in Epidemiology (STROBE) reporting guideline, and informed consent was waived because data were deidentified.

### Study Design

We used a difference-in-differences design that leveraged county-by-year variation in SBHC adoption. The study period was one of substantial growth in SBHCs.^1^ Expansion occurred in all regions of the country (eFigure 1 in Supplement 1). The design was implemented with regressions that controlled for county to account for factors that were consistent over time (eg, persistent poverty rates and health system infrastructure) and year to account for trends common across counties (eg, macroeconomic fluctuations).

### Data

Data came from the 1997 to 2018 National Health Interview Survey (NHIS).^9,10^ The date range 1997 to 2018 was chosen because it was consistent with the variation in SBHC adoption described later and because those years of the NHIS are comparable across time. The NHIS is a nationally representative repeated cross-sectional survey that collects in-person interviews. The survey, sponsored by the National Center for Health Statistics (NCHS), typically achieves a 70% response rate.

We used a restricted version of the NHIS that included county identifiers to merge individual NHIS records with county-by-year indicators of SBHC availability. We measured SBHC adoption at the county level because sample sizes prohibited analysis at lower levels. About 40% of school-aged children in counties with at least 1 SBHC resided in school districts with an SBHC. The mean number of centers in counties with a center was 4.7. While the county-based approach had disadvantages in terms of precision, it also ensured that estimates were not biased by selective migration within a county (roughly 65% of US movers in our study period moved within county).^11^ It also allowed us to capture spillovers. For example, when an SBHC opens it might mitigate clinician shortages in neighboring communities that do not have a center.

Information about SBHCs at the county-year level came from the National Census of School Based Health Centers conducted by the School-Based Health Alliance (SBHA).^2,12^ The Census is a triennial survey of all SBHCs. It gathers information about services, staffing, and students served. Centers are identified from SBHA state affiliates or memberships, state governments, and convention registrations. The most recent Census obtained a 90% response rate.

The Census was used to identify county-years in which at least 1 SBHC was operational (vs none). We used questions about the opening year from the most recent wave (2016-2017) to retrospectively identify if a county had a center in a specific year. Because the absence of a center during the 2016 to 2017 school-year wave could reflect inaccuracies or nonresponse, we required that county-years classified as not having a center based on the wave during the 2016 to 2017 school year also did not have a center in any prior wave (ie, 1998-2014). Approximately 8% of counties identified as not having a center during the 2016 to 2017 year did have a center in a previous wave and were excluded.

### Analytic Sample and Subgroups

We made several sample exclusions to improve internal validity. Analyses were confined to centers that offered comprehensive primary care. Less than 10% of centers offer single services and could have distinct impacts.^1^ We excluded the 10% of counties that we identified as having a center but were missing information on opening date. We restricted the sample to counties that were observed in every year of the NHIS to ensure a similar set of counties across years. We omitted children living in the 200 counties that adopted a center before 2003 to ensure that we observed at least 6 years of preadoption outcomes in all adoption counties. This improved our ability to assess differences in preimplementation outcome trends, an important test of internal validity. It also ensured that counties that had a center in every year never served as the comparison group (a comparison that can bias difference-in-differences).^13^ Additionally, we omitted children in counties that adopted a center after 2013, which allowed us to assess postimplementation outcomes for a minimum of 6 years in all included counties and up to 16 years in some counties.

Analyses focused on children aged 5 to 17 years. Most analyses pertained to children with family incomes below 200% of the federal poverty line (FPL) because SBHCs target children who are disadvantaged and face the greatest access burdens.^14,15,16,17^ However, we also included children from higher income families (≥200% FPL) to evaluate if SBHCs were associated with reductions in income-based disparities. We estimated associations among children from low-income families separately by sex and age group (ie, 5-10 years, 11-13 years, and 14-17 years) because health care needs could vary by subgroup. We omitted children who were missing information on the outcomes or covariates (about 4% of the unweighted sample). We used multiple imputed income measures produced by NCHS.^18^ More information on missingness is provided in the eTable 1 in Supplement 1.

### Outcomes

We examined 3 categories of dichotomous outcomes. Access measures were chosen that we expected would be sensitive to SBHC adoption and included having a usual source of care, experiencing a cost barrier (ie, needing, but unable to afford medical, vision, dental, or pharmaceutical services) or a noncost barrier (ie, delaying care due to transportation, wait times, appointment availability, scheduling conflicts, or inability to contact a clinician). We examined insured status because centers might help facilitate insurance take-up. Utilization measures included ambulatory care variables that captured a comprehensive set of services—visiting a physician or other health professional, eye doctor, dentist, or mental health clinician in the last 12 months. SBHCs might have direct associations with eye doctor visits, dentists, and mental health clinicians in centers that offer those services and could have indirect associations in centers that do not via referral. Health outcomes included general reported health (ie, very good or excellent vs good, fair, or poor) and if the child missed 4 or more school days due to illness. We chose health outcomes with measurements that did not depend on interaction with the health system (eg, diagnoses) because interpreting such outcomes is ambiguous (ie, diagnoses could increase with improved access or decrease with improved health).

### Covariates

Covariates included characteristics that influence the health and service use of children, including age (5-10 years, 11-13 years, 14-17 years), sex (male, female), highest education in the household (less than high school, high school, some college or more), household marital status (married vs not), and household employment (any employed adult vs none). We included self-reported race and ethnicity (Hispanic, non-Hispanic Black, non-Hispanic White, and non-Hispanic other). The other category included Asian, Pacific Islander, and Native American, which were combined due to small sample sizes.

### Statistical Analysis

We implemented the difference-in-differences using linear probability models. Sensitivity analyses investigated if we came to similar conclusions using logistic regression. In addition to the SBHC adoption indicator, regressions controlled for county, survey year, and the covariates described above.

We used the 2-stage difference-in-differences regression approach.^19^ The typical approach to estimating difference-in-differences, TWFE, can be biased when the timing of adoption varies.^13,20^ Two-stage difference-in-differences overcomes this by regressing outcomes on county indicators, year indicators, and covariates in the untreated sample and then generating residuals for the full sample. The residuals are then regressed on the SBHC adoption indicator and covariates. The approach was implemented in a generalized methods of moments framework to measure sampling variances from both stages using standard errors clustered on county. We report *P* values that have been adjusted for multiple comparisons using the sharpened *q* value method.^21,22^ Two-sided tests, conducted in Stata version 16 (StataCorp), were considered significant at *P* ≤ .05. All estimates were weighted by sampling weights.

We conducted subgroup analyses by estimating stratified regressions by poverty group (below 200% FPL and above 200% FPL), and by age and sex in the low-income sample. To assess associations of SBHC adoption with income-based disparities, we estimated models that interacted the SBHC indicator with a poverty group indicator. Using the coefficients, we calculated adjusted differences in outcomes by poverty group, for children with and without access to an SBHC. The disparities analysis used TWFE regressions. We provide a rationale for that choice in the eAppendix in Supplement 1 and show that the 2-stage approach suggests similar conclusions. We limited the disparities analysis to outcomes that were statistically associated with SBHC availability in the low-income sample.

Finally, we estimated event study models using the 2-stage procedure. These analyses replaced the binary adoption indicator with a series of time until adoption indicators, where the year prior to adoption was the reference category. The models assessed if associations emerged prior to adoption, suggesting a violation of the parallel trends assumption. Data were collected from January 1997 to December 2018, and data were analyzed from January 2020 to July 2023.

We conducted sensitivity analyses to determine if we came to consistent results with reasonable alterations in approach. Because state-level Medicaid or Children’s Health Insurance Program eligibility expansions may have increased children’s access to care, we estimated models that included state-by-year income eligibility limits.^23^ We estimated models that removed all covariates except for county and year, relaxed the requirement that counties must appear in all years, explored alternatives to removing counties that adopted a center prior to 2003 or after 2013, removed the sample weights, and estimated 2-way fixed effects (TWFE) using linear and logistic regressions.

## Results

This study included an unweighted 12 624 children from low-income families and 24 631 children from higher-income families. The weighted percentages of children in low-income families who resided in an SBHC adoption county included 50.0% (95% CI, 48.2%-51.7%) aged 5 to 10 years, 22.2% (95% CI, 20.8%-23.7%) aged 11 to 13 years, and 27.8% (95% CI, 26.2%-29.5%) aged 14 to 17 years. The weighted percentages of the race and ethnicity of these children included 36.7% (95% CI, 33.6%-39.9%) Hispanic children, 25.2% (95% CI, 22.7%-27.8%) non-Hispanic Black children, and 30.6% (95% CI, 27.9%-33.4%) non-Hispanic White children. The weighted percentages of children in low-income families who resided in counties that never adopted SBHCs included 50.1% (95% CI, 48.4%-51.7%) aged 5 to 10 years, 22.1% (95% CI, 2.9%-23.4%) aged 11 to 13 years, and 27.8% (95% CI, 26.4%-29.2%) aged 14 to 17 years. The weighted percentages of the race and ethnicity of these children included 20.7% (95% CI, 18.2%-23.5%) Hispanic children, 22.4% (95% CI, 2.0%-25.0%) non-Hispanic Black children, and 52.9% (95% CI, 50%-55.8%) non-Hispanic White children. Additional demographic data can be found in Table 1, and a sample flow diagram is provided in eFigure 2 in Supplement 1.

**Table 1.** Population Characteristics by School-Based Health Centers Exposure Status, Children From Low-Income Families, 1997-2018 National Health Interview Survey^**a**^

Characteristics	Adoption counties		Never adopted counties		*P* value
	Weighted count	Weighted % (95% CI)	Weighted count	Weighted % (95% CI)	
Age, y					
5-10	20 824 247	50.0 (48.2-51.7)	25 389 863	50.1 (48.4-51.7)	.62
11-13	9 255 944	22.2 (20.8-23.7)	11 212 425	22.1 (2.9-23.4)	
14-17	11 593 586	27.8 (26.2-29.5)	14 103 323	27.8 (26.4-29.2)	
Race or ethnicity					
Hispanic	15 306 756	36.7 (33.6-39.9)	10 504 240	20.7 (18.2-23.5)	<.001
Non-Hispanic Black	10 496 958	25.2 (22.7-27.8)	11 348 576	22.4 (2.0-25.0)	
Non-Hispanic White	12 731 527	30.6 (27.9-33.4)	26 825 779	52.9 (50-55.8)	
Non-Hispanic Other^b^	3 138 536	7.5 (6.0-9.4)	2 021 893	4.0 (3.4-4.7)	
Sex					
Female	20 679 097	49.6 (48.1-51.2)	25 244 389	49.8 (48.4-51.2)	.67
Male	20 994 680	50.4 (48.8-51.9)	25 461 222	5.2 (48.8-51.6)	
Poverty level, % FPL					
0-99	19 446 384	46.7 (44.7-48.6)	21 038 235	41.5 (39.5-43.5)	<.001
100-149	11 590 264	27.8 (26.3-29.4)	14 862 796	29.3 (27.8-3.9)	
150-199	10 637 129	25.5 (23.9-27.2)	14 804 580	29.2 (27.6-3.8)	
Household education					
Less than high school	9 272 039	22.3 (20.7-23.9)	8 301 547	16.4 (14.9-18.1)	<.001
High school	12 495 908	30.0 (28.4-31.8)	16 082 035	31.8 (3.2-33.5)	
Some college or more	19 836 549	47.7 (45.6-49.7)	26 160 371	51.8 (49.7-53.8)	
Household marital status					
Married	22 774 325	54.6 (52.6-56.7)	27 056 249	53.4 (51.6-55.1)	.82
Household employment					
Working	34 431 521	82.6 (81.1-84.0)	41 629 108	82.1 (8.7-83.4)	.41
Unweighted sample size	6321	NA	6303	NA	NA

Abbreviations: FPL, federal poverty level; NA, not applicable.

^a^ Data are from the 1997 to 2018 National Health Interview Survey merged with school-based health center adoption indicators from the Census of School-Based Health Centers.^9,12^ Estimates pertain to children in families below 200% of poverty. The *P* value is for the association between a given characteristic and adoption status. All estimates are weighted to represent person-years, and 95% CIs are constructed using a logit transformation, so the end points are bounded by 0 and 100.

^b^ Other race includes Asian, Pacific Islander, and Native American.

**Figure 1.**
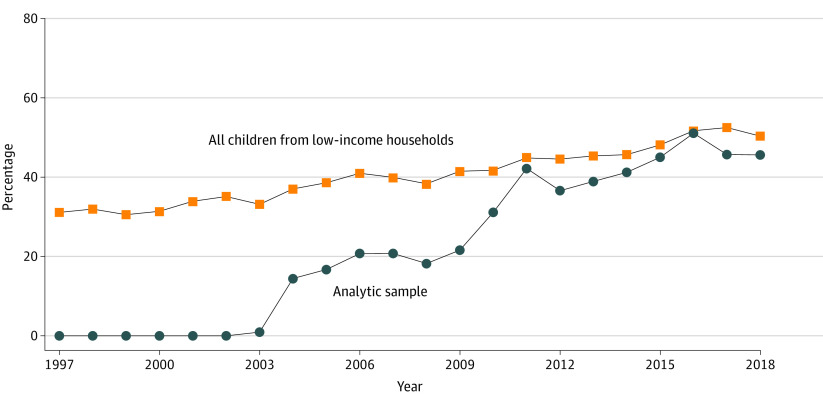
Percentage of School Age Children in Low-Income Families With Access to a School-Based Health Center (SBHC), 1997-2018 National Health Interview Survey (NHIS) Data are from the 1997 to 2018 NHIS merged with SBHC adoption indicators from the Census of School-Based Health Centers.^9,12^ The analytic sample includes children from low-income families residing in counties consistently observed in every NHIS year that either obtained a center between 2003 and 2013 or never obtained a center.

Figure 1 describes the weighted percentage of all children from low-income families and the percentage of children from low-income families in the analytic population who lived in a county with an SBHC. Overall, the percentage of children from low-income families who lived in a county with an SBHC increased from 31.1% to 50.3% over the study period. By construction, there were no children in the analytic sample who lived in a county with an SBHC prior to 2003 and exposure grew to 45.6% by the end of the study period. In an average year, 22.3% of children from low-income families in the analytic population lived in a county with an SBHC.

Table 1 describes analytic population characteristics of children from low-income families in counties that adopted a center vs children in counties that never obtained a center. The groups were balanced on age, sex, household marital status, and household employment. The groups were not balanced by race and ethnicity, poverty, and household education (Table 1). This is consistent with the adoption of centers in disadvantaged counties. To the extent that relative disadvantage was consistent over time, our models accounted for it by controlling for county. Consistent with that pattern, eTable 2 in Supplement 1 shows that conditional on year and county, SBHC adoption is not associated with race, poverty or household education. eTable 3 in Supplement 1 provides descriptive statistics for children from higher income families, and eTable 4 in Supplement 1 compares children included and excluded from the analytic sample.

Table 2 displays the association of SBHC adoption with the outcomes from the difference-in-differences regressions. In the low-income population, SBHCs were associated with an increase of 6.4 percentage points (95% CI, 3.2 to 9.6 percentage points; *P* < .001) in having a dental visit in the last 12 months, an increase of 8.0 percentage points (95% CI, 4.5 to 11.5 percentage points; *P* < .001) in having a usual source of care and a 5.2 percentage point increase in being insured (95% CI, 1.2 to 9.2 percentage points; *P* = .03). Associations were not statistically significant for general health professional visits (2.7 percentage points; 95% CI, −0.9 to 6.3 percentage points; *P* = .15), eye doctor visits (3.5 percentage points; 95% CI, −0.2 to 7.2 percentage points; *P* = .10), mental health visits (−0.8 percentage points; 95% CI, −3.6 to 9.6 percentage points; *P* = .26), financial barriers (−2.9 percentage points; 95% CI, −6.1 to 0.3 percentage points; *P* = .10); nonfinancial barriers (1.1 percentage points; 95% CI, −2.2 to 4.4 percentage points; *P* = .26), self-reported health (−3.4 percentage points; 95% CI, −3.4 to 0.3 percentage points; *P* = .10), and missed school days (0.4 percentage points; 95% CI, −4.2 to 5.0 percentage points; *P* = .35). In the higher income population, all associations were smaller than 1.4 percentage points in absolute value and *P* values exceeded .95 for all outcomes (Table 2).

**Table 2.** Two-Stage Difference-in-Differences Estimates by Income Group, 5-17 Year Olds^**a**^

Variable	<200% Federal poverty level (n = 12 264)			≥200% FPL (n = 24 631)		
	Preadoption (%)	Estimate (95% CI)^b^	Adjusted *P* value^c^	Preadoption (%)	Estimate (95% CI)^b^	Adjusted *P* value^c^
Access and use						
Doctor or health professional visit	81.9	2.7 (−0.9 to 6.3)	.15	90.4	0.3 (−1.8 to 2.3)	≥.95
Eye doctor visit	18.7	3.5 (−0.2 to 7.2)	.10	26.7	−1.4 (−3.9 to 1.0)	≥.95
Dentist visit	70.0	6.4 (3.2 to 9.6)	≤.001	88.0	1.3 (−0.9 to 3.5)	≥.95
Mental health visit	7.3	−0.8 (−3.6 to 2.0)	.26	6.3	0.1 (−1.8 to 2)	≥.95
Usual source of care	86.6	8.0 (4.5 to 11.5)	≤.001	96.1	−1.0 (−2.1 to 0.2)	≥.95
Insured	79.7	5.2 (1.2 to 9.2)	.03	94.1	0.6 (−0.8 to 2)	≥.95
Any financial barrier	16.1	−2.9 (−6.1 to 0.3)	.10	6.4	−0.1 (−1.6 to 1.4)	≥.95
Any nonfinancial barrier	12.2	1.1 (−2.2 to 4.4)	.26	7.8	0.5 (−1.1 to 2.2)	≥.95
Health						
Excellent or very good health	74.9	−3.4 (−7.1 to 0.3)	.10	89	0.4 (−1.4 to 2.3)	≥.95
>4 Missed school days	32.2	0.4 (−4.2 to 5.0)	.35	31.1	−0.6 (−3.3 to 2.0)	≥.95

^a^ Data are from the 1997 to 2018 National Health Interview Survey and the Census of School-Based Health Centers.^9,12^ The sample includes only counties that are consistently observed in every year and either obtained a center between 2003 and 2013 or never obtained a center. All visit variables are over 12 months prior to interview. Models control for age, sex, race and ethnicity, household education, household marriage, household employment, year, and county. Preadoption means are for children in adoption counties, prior to school-based health center introduction.

^b^ Estimates are 2-stage difference-in-differences estimates and are expressed as percentage points, and 95% CIs are derived from robust standard errors clustered on counties that have not been adjusted for multiple comparisons.

^c^ The adjusted *P* value adjust for multiple comparisons using sharpened *q* values. All estimates are weighted.

Results from the event-study are presented in eFigure 3 in Supplement 1. We found little indication that the associations observed in Table 2 were emerging prior to the adoption of an SBHC, which supports the study design. Sensitivity analyses suggested that results were robust to including Medicaid eligibility limits as a control variable, excluding demographic covariates, relaxing the sample criteria, omitting sampling weights, and estimating 2-way fixed models using linear probability or logistic specifications (eFigure 4 and eTable 5 in the Supplement).

**Figure 2.**
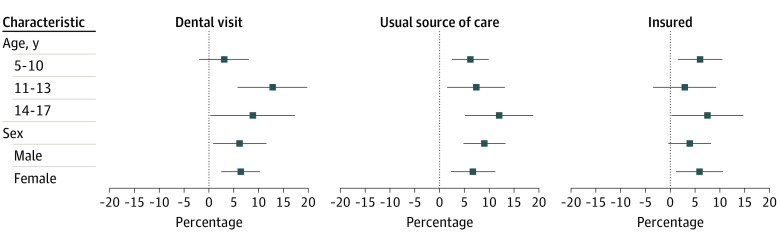
Difference-in-Differences Estimates by Subgroup, Children From Low-Income Families Data are from the 1997 to 2018 National Health Interview Survey merged with SBHC adoption indicators from the Census of School-Based Health Centers.^9,12^ Estimates are from stratified 2-stage difference-in-difference regressions. Error bars denote 95% CIs.

Figure 2 describes associations between SBHC adoption within the low-income population with dental visits, usual source of care, and insured status by age group and sex. While there was some indication that associations with dental visits were larger for middle school-aged children, in general the associations were consistent across group.

**Figure 3.**
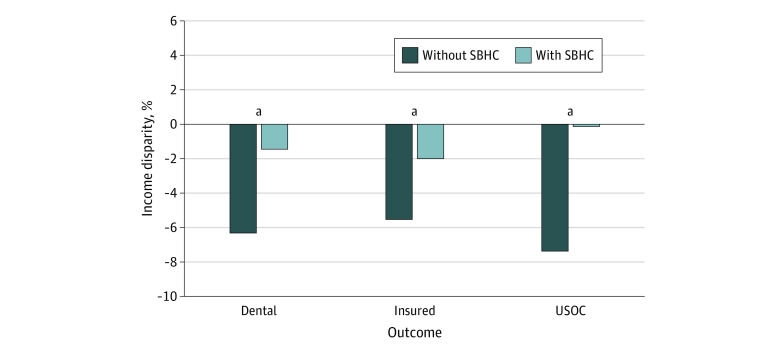
The Association of School-Based Health Centers (SBHCs) With Income-Based Disparities Data are from the 1997 to 2018 National Health Interview Survey merged with SBHC adoption indicators from the Census of School-Based Health Centers.^9,12^ Estimates are derived from ordinary least-squares difference-in-difference regressions with income group interaction terms that allow us to recover the disparity with and without SBHCs. We show in the eAppendix in Supplement 1 that the interaction coefficients are similar in the 2-stage and ordinary least-squares models. USOC indicates usual source of care. ^a^The reduction in disparity is statistically significant at *P* ≤ .05.

Income-based disparities with and without access to an SBHC for dental visits, usual source of care, and insurance coverage are displayed in Figure 3. SBHCs reduced income-based disparities in dental visits (by 4.9 percentage points; 95% CI, 2.0 to 7.7 percentage points), insured status (by 3.5 percentage points; 95% CI, 1.3 to 5.7 percentage points) and usual source of care (by 7.2 percentage points; 95% CI, 5.4 to 9.1 percentage points). This represents a relative reduction of 76%, 63%, and 98%, respectively. Detailed results are shown in eTable 6 in Supplement 1.

## Discussion

The findings of this study suggest that SBHCs increase dental visits, having a usual source of care, and having insurance. Associations were concentrated among children from low-income families, and there were no associations among children from higher income families. This suggests that there may be a role for SBHCs in reducing income-based disparities. Our estimates suggest that SBHCs can substantially reduce income-based disparities in insurance coverage and dental visits and nearly eliminate them for usual source of care. Finding that SBHCs increase insurance coverage suggests that SBHCs play an important role in helping families navigate the administrative burdens that discourage enrollment in public programs.^3,24^

Results for dental access are encouraging since tooth decay is among the most common chronic diseases of childhood.^25^ SBHCs could promote school-based dental visits for children with access to the 28% of centers that offer these services^1^ or they could increase visits indirectly through referral. Only about half of the children from low-income families who are enrolled in Medicaid visit the dentist regularly, despite the fact that all programs cover dental services.^25^ SBHCs might reduce these gaps. More work is needed.

Although there is a strong conceptual case that SBHCs might increase use by reducing nonfinancial barriers, we did not find evidence of that association. This might suggest that SBHCs instead succeed by promoting services that children and families are unaware of or underestimate the value of obtaining. This could have implications for the recent growth in SBHC telehealth and future work is needed.^26^ While telehealth has advantages, it might reduce capacity to promote services.^27^

Consistent with previous studies we did not find evidence that SBHCs were associated with general health status.^5,16^ This could reflect limitations in the measurement of self-reported health or the long time spans that are often required for pediatric population health interventions to manifest in health outcomes.^28,29^ However, previous research has found that centers do improve outcomes for children with specific conditions, such as asthma.^30,31^ Continued research is needed on what dimensions of health are most sensitive to SBHCs and to the longer-term benefits that SBHCs might have.

Our results agree with existing research that suggests that SBHCs are an effective model of delivery.^5,7,8,32,33,34,35^ However, our study has particular strengths and overcomes many of the limitations encountered in previous research.^5,6^ Our quasi-experimental design accounts for observed and unobserved time-invariant factors that sort centers into communities, such as persistently high poverty rates or existing health system resources that are needed to launch a center.

### Limitations

This study has limitations. It possible that the timing of adoption was associated with factors that affected outcomes. However, these risks are likely minimal because associations did not begin to emerge until after adoption, adoption was not associated with changes in population composition, and sensitivity analyses suggested consistent results.

Due to sample size constraints, county was the lowest level of geography we could use. County adoption overstates the exposure that children actually faced in their school districts, and our estimates likely represent a lower bound. However, our design also avoided potential bias from within county migration, and it allowed for SBHC spillovers in neighboring communities.

By basing our study on the national expansion of SBHCs, our estimates reflect the average experience of adoption and are useful for developing expectations about the benefit of further expansions. However, there is heterogeneity across SBHCs. Further work is needed on best practices.

Finally, we removed counties that the Census for the 2016 to 2017 school year indicated did not have a center but were found to have one in the 1998 to 2014 waves. This ensured that the comparison group only included counties that we were highly confident never had a center. However, if excluded counties had centers that closed prior to the 2016 to 2017 school year because they were less successful, our results might be upwardly biased.

## Conclusions

This study suggests that SBHCs are effective at improving access and use of services among children from low-income families. Our findings also indicated that SBHCs have the potential to reduce income-based disparities. Future investments in SBHC delivery will likely produce additional benefits.

## Notes

Supplement 1. **eFigure 1.** County-Level SBHC Adoption, 1998-2017 **eFigure 2.** Sample Flow Diagram **eTable 1.** Percentage Missing, Low-Income Children, 1997-2018 Public Use NHIS **eTable 2.** Balancing Test (2-Stage Difference-in-Differences), Low-Income Children **eTable 3.** Population Characteristics by SBHC Exposure Status, Higher-Income Children, 1997-2018 National Health Interview Survey **eTable 4. **Population Characteristics by Sample, Low-Income Children, 1997-2018 National Health Interview Survey **eFigure 3.** Two-Stage Event Study Estimates **eFigure 4.** Sensitivity Analysis, Low-Income Children **eTable 5.** Two-Way Fixed Effects, Logistic Regression, Low-Income Children Aged 5-17 Years **eTable 6.** The Association of SBHCs and Income-Based Disparities

Supplement 2.
Data Sharing Statement
