# Errors in Table 1 and the Results

**DOI:** 10.1001/jamanetworkopen.2023.40621

**Published:** 2023-10-19

In the Original Investigation titled “School-Based Health Centers, Access to Care, and Income-Based Disparities,”^1^ published September 18, 2023, there were transcription errors in Table 1 affecting several percentages and their confidence intervals in the column reporting weighted percentages of counties that did not adopt school-based health centers. There were also 2 transcription errors in the text affecting the confidence intervals of the percentages. This article has been corrected.^1^
