# Supplementary material for: School-Based Health Centers, Access to Care, and Income-Based Disparities
Source: JAMA Netw Open. 2023 Sep 18;6(9):e2334532. doi: 10.1001/jamanetworkopen.2023.34532 (PMC10507491; doi:10.1001/jamanetworkopen.2023.34532)
Supplement: Supplement 1. — eFigure 1. County-Level SBHC Adoption, 1998-2017 eFigure 2. Sample Flow Diagram eTable 1. Percentage Missing, Low-Income Children, 1997-2018 Public Use NHIS eTable 2. Balancing Test (2-Stage Difference-in-Differences), Low-Income Children eTable 3. Population Characteristics by SBHC Exposure Status, Higher-Income Children, 1997-2018 National Health Interview Survey eTable 4. Population Characteristics by Sample, Low-Income Children, 1997-2018 National Health Interview Survey eFigure 3. Two-Stage Event Study Estimates eFigure 4. Sensitivity Analysis, Low-Income Children eTable 5. Two-Way Fixed Effects, Logistic Regression, Low-Income Children Aged 5-17 Years eTable 6. The Association of SBHCs and Income-Based Disparities [file jamanetwopen-e2334532-s001.pdf]

Supplemental Online Content

Boudreaux M, Chu J, Lipton BJ. School-based health centers, access to care, and income-based disparities. *JAMA Netw Open*. 2023;6(9):e2334532. doi:10.1001/jamanetworkopen.2023.34532

eFigure 1. County-Level SBHC Adoption, 1998-2017

eFigure 2. Sample Flow Diagram

eTable 1. Percent Missing, Low-Income Children, 1997-2018 Public Use NHIS

eTable 2. Balancing Test (Two-Stage Difference-in-Differences), Low-Income Children

eTable 3. Population Characteristics by SBHC Exposure Status, Higher-Income Children, 1997-2018 National Health Interview Survey

eTable 4. Population Characteristics by Sample, Low-Income Children, 1997-2018 National Health Interview Survey

eFigure 3. Two-Stage Event Study Estimates

eFigure 4. Sensitivity Analysis, Low-Income Children

eTable 5. Two-Way Fixed Effects, Logistic Regression, Low-Income Children Age 5-17 Years

eTable 6. The Association of SBHCs and Income-Based Disparities

This supplemental material has been provided by the authors to give readers additional information about their work.

© 2023 Boudreaux M et al. *JAMA Network Open.*

School-Based Health Centers, Access to Care, and Income-Based Disparities

Supplemental Appendix

eFigure 1 displays county-level adoption of an SBHC between 1998 and 2017. Never treated counties were never identified as having a wave in any Census wave. Always treated counties are counties that had a center than opened prior to 1998. Adoption counties obtained their first center in the 1998-2017 period. Missing counties are known to have a center, but their opening dates are not known. The map shows that adoption was distributed across all areas of the United States.

eFigure 1. County-Level SBHC Adoption, 1998-2017

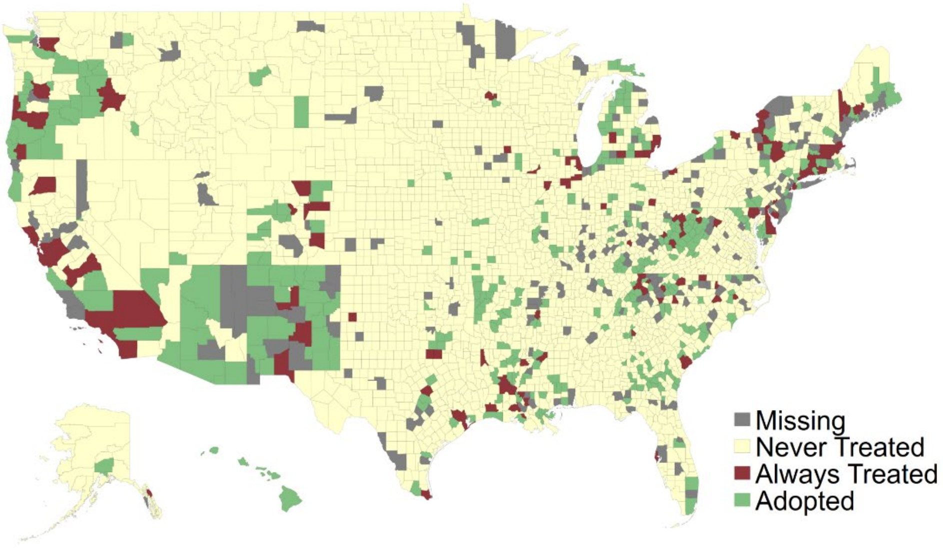

Source: 1998-2017 National School-Based Health Center Census

© 2023 Boudreaux M et al. *JAMA Network Open.*

eFigure 2. Sample Flow Diagram

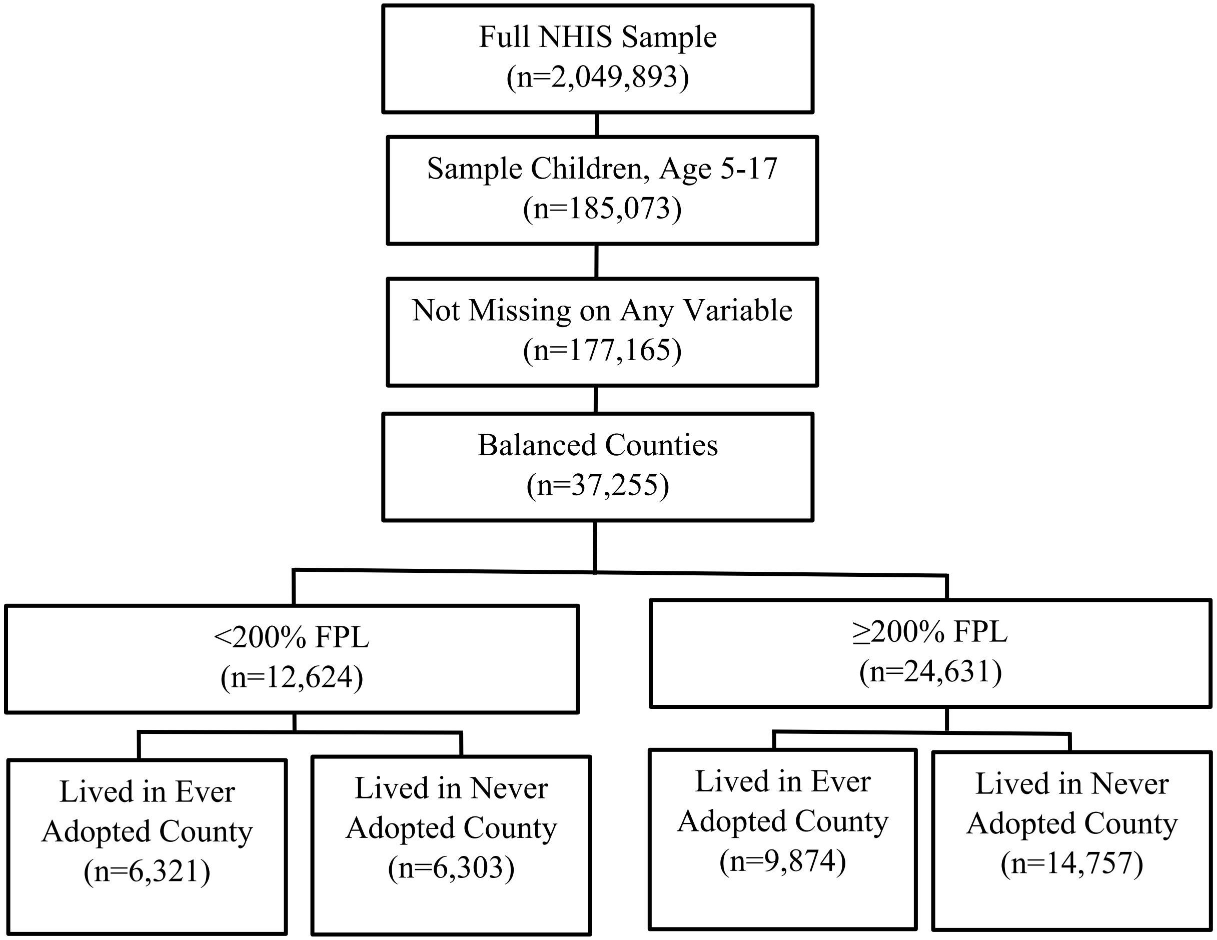

Source/Notes. 1997-2018 National Health Interview Survey. Balanced county means observed in every year. FPL is federal poverty level. Sample children are children selected for additional questions in the NHIS. All sample sizes are unweighted.

© 2023 Boudreaux M et al. *JAMA Network Open.*

eTable 1 reports the unweighted missing rate due to item non-response for all key variables for low-income children (<200% FPL) observed in the public use NHIS sample child files during our study period. Due disclosure rules, the missing rate for individual variables is not releasable for our analytic sample (due to small cell sizes). However, the results from the public use file described below provide useful information about patterns of missingness, which are low for all key variables. NCHS fully imputes age, sex and race/ethnicity to facilitate weighting. They also provide multiply imputed income and poverty measures, that we use in this study. Details on the method are provided in the citations provided in the main text.

eTable 1. Percent Missing, Low-Income Children, 1997-2018 Public Use NHIS

	% Missing
Age	0
Race/Ethnicity	0
Sex	0
Poverty Level	0
Household Education	0.25
Household Marital Status	0.01
Household Employment	0
Doctor/Health Professional Visit	1.34
Eye Doctor Visit	0.55
Dentist Visit	1.48
Mental Health Visit	0.46
Usual Source of Care	0.21
Insured	0.58
Any Financial Barrier	0.07
Any Non-Financial Barrier	0.19
Excellent/Very Good Health	0.05
4+ Missed School Days	2.97

Source: 1997-2018 National Health Interview Survey, Public Use Files. % Missing is unweighted. Age, sex, race/ethnicity, and poverty is multiply imputed by NCHS.

© 2023 Boudreaux M et al. *JAMA Network Open.*

eTable 2 describes the results of a balancing test in which indicators for being in poverty, Non-Hispanic white, and living in household where the maximum education is high school or less are used as outcomes in a difference-in-difference analysis. The regressions, implemented with the two-stage approach described in the text, control for county and year effects. The point of this test is determine if covariates that were found to be statistically associated with *ever* adopting an SBHC (Table 1) were correlated with the adoption itself. We failed to find evidence of such an association. The results suggest that SBHC adoption was not correlated with changes in population composition that might bias our main results. Such correlations might emerge, for example, if the adoption of a center induced changes in migration patterns or if SBHCs were adopted at a time the co-occurred with broader macroeconomic fluctations.

eTable 2. Balancing Test (Two-Stage Difference-in-Differences) , Low-Income Children

	Est.	95% CI	P-Value
Poverty	1.3	(-3.8, 6.4)	0.607
High School or Less	-1.1	(-7.9, 5.6)	0.739
Non-Hispanic White	1.3	(-4.1, 6.7)	0.629

Source: 1997-2018 National Health Interview Survey merged with county-level SBHC adoption indicators from the National Census of School-Based Health Centers.

eTable 3 reports descriptive statistics for higher income children (at or above 200% FPL) living in counties that ever adopted an SBHC versus children living in counties that adopted, per our analytic sample definitions. Similar to Table 1 in the main manuscript, the statistical tests suggest that the sample is not balanced on race/ethnicity and household education. Unlike the low-income sample in Table 1, higher income children are also not balanced on household marital status.

eTable 3. Population Characteristics by SBHC Exposure Status, Higher-Income Children, 1997-2018 National Health Interview Survey

	Adoption Counties			Never Adopted Counties			P-Value
	Weighted Count	Percent	95% CI	Weighted Count	Percent	95% CI	

© 2023 Boudreaux M et al. *JAMA Network Open.*

**Age**							0.139
5-10y	34,922,258	44.8	(43.6, 45.9)	111,278,420	43.8	(42.7, 44.8)	
11-13y	18,656,995	23.6	(22.6, 24.5)	61,586,799	24.2	(23.4, 25.1)	
14-17y	25,154,515	31.7	(30.6, 32.7)	84,770,462	32.0	(31.2, 32.8)	
**Race/Ethnicity**							<0.001
Hispanic	9,874,986	13.9	(12.7, 15.2)	14,995,320	5.8	(5.1, 6.5)	
Non-Hispanic Black	8,709,033	12.4	(10.9, 14)	17,436,292	8.3	(7.6, 9.1)	
Non-Hispanic White	54,010,375	64.7	(62.6, 66.8)	216,298,579	81.4	(80.2, 82.5)	
Non-Hispanic Other[a]	6,103,716	9.0	(8, 9.9)	8,894,797	4.5	(4, 4.9)	
**Sex**							0.743
Female	38,577,876	49.2	(48.1, 50.4)	125,698,795	48.3	(47.4, 49.2)	
Male	40,155,892	50.8	(49.6, 51.9)	131,936,886	51.7	(50.8, 52.6)	
**Household Education**							<0.001
Less than High School	1,901,757	2.5	(2.1, 2.9)	5,431,544	1.7	(1.4, 2)	
High School	10,200,675	11.9	(10.8, 13.1)	40,677,342	12.7	(11.8, 13.5)	
Some College or More	66,499,629	85.6	(84.3, 86.9)	210,879,330	85.6	(84.6, 86.6)	
**Household Marital Status**							<0.001
Married	65,745,475	83.2	(82.1, 84.3)	220,345,472	85.2	(84.5, 86)	
**Household Employment**							0.932
Working	77,425,099	98.4	(98.1, 98.7)	25,3319,946	98.4	(98.2, 98.7)	

Source: 1997-2018 National Health Interview Survey merged with SBHC adoption indicators from the National School-Based Health Center Census. Note: Estimates pertain to children in families at or above 200% of poverty. The p-value is for the association between a given characteristic and adoption status. All estimates are weighted. 95% CI are 95% confidence intervals.
(a) Other race includes Asian, Pacific Islander, and Native American.

© 2023 Boudreaux M et al. *JAMA Network Open.*

Our main analytic sample sets several exclusion/inclusion rules to improve internal validity. The major rules included only including children that live in counties that were consistently observed in every year of the NHIS (to create a balanced panel), removing children that adopted a center prior to 2003 (so that every adopting county had at least 6 pre-period years), removing observations in counties that adopted after 2014 (so that all observations living in adoption counties experienced at least 6 years of post-adoption time), and removing counties that did not have a center in the 2016-17 Census, but were found to have one in a prior Census wave. These rules optimize the internal validity of our sample, but the sample only generalizes to our sample inclusion/exclusion rules and the target population. For completeness, eTable 4 compares low-income children that meet the sample inclusion/exclusion rules and children that do not. Overall, the included sample is more disadvantaged than the excluded sample. Low-income children in the analytic sample have a different racial/ethnic composition compared to excluded children (less likely to be non-Hispanic White), they are more likely to live in poverty, and have lower household educational attainment. With this caviate in mind, later in the eAppendix we show that we can relax some of the key sample inclusion/exclusion rules and we come to similar conclusions.

© 2023 Boudreaux M et al. *JAMA Network Open.*

eTable 4. Population Characteristics by Sample, Low-Income Children, 1997-2018 National Health Interview Survey

	Excluded from Analytic Sample			Included in Analytic Sample			P-Value
	Weighted Count	Percent	95% CI	Weighted Count	Percent	95% CI	
**Age**							0.090
5-10y	46,214,110	50.0	(48.8, 51.3)	186,164,767	48.6	(48, 49.1)	
11-13y	20,468,369	22.2	(21.2, 23.1)	87,006,742	22.7	(22.3, 23.1)	
14-17y	25,696,909	27.8	(26.7, 28.9)	110,082,707	28.7	(28.2, 29.2)	
**Race/Ethnicity**							<0.001
Hispanic	25,810,996	27.9	(26, 29.9)	124,796,565	32.6	(31.6, 33.5)	
Non-Hispanic Black	21,845,534	23.6	(21.9, 25.4)	84,003,243	21.9	(21.1, 22.8)	
Non-Hispanic White	39,557,306	42.8	(40.8, 44.9)	153,032,798	39.9	(39, 40.9)	
Non-Hispanic Other[a]	5,160,429	5.6	(4.7, 6.4)	21,368,750	5.6	(5.1, 6)	
**Sex**							0.209
Female	45,923,486	49.7	(48.7, 50.7)	187,738,993	49.0	(48.5, 49.5)	
Male	46,455,902	50.3	(49.3, 51.3)	195,515,223	51.0	(50.5, 51.5)	
**Poverty Level**							0.002
0-99% FPL	40,484,619	43.8	(42.4, 45.2)	176,037,876	45.9	(45.3, 46.6)	
100-149% FPL	26,453,060	28.6	(27.5, 29.7)	110,007,856	28.7	(28.2, 29.2)	
150-199% FPL	25,441,709	27.5	(26.4, 28.7)	97,208,484	25.4	(24.9, 25.9)	
**Household Education**							<0.001
Less than High School	17,573,586	19.1	(18, 20.1)	86,070,884	22.5	(21.9, 23.1)	
High School	28,577,943	31.0	(29.8, 32.2)	123,711,510	32.4	(31.8, 32.9)	

© 2023 Boudreaux M et al. *JAMA Network Open.*

Some College or More	45,996,920	49.9	(48.5, 51.4)	172,512,572	45.1	(44.5, 45.8)	
**Household Marital Status**							0.322
Married	49,830,574	53.9	(52.6, 55.3)	209,588,177	54.7	(54, 55.3)	
**Household Employment**							0.223
Working	76,060,629	82.3	(81.3, 83.3)	312,936,124	81.7	(81.2, 82.1)	

Source: 1997-2018 National Health Interview Survey merged with SBHC adoption indicators from the National School-Based Health Center Census. Note: Estimates pertain to children in families below 200% of poverty. The p-value is for the association between a given characteristic and sample component. All estimates are weighted. 95% CI are confidence intervals. (a) Other race includes Asian, Pacific Islander, and Native American.

© 2023 Boudreaux M et al. *JAMA Network Open.*

eFigure 3 plots coefficients from the event-study analysis described in the main text. The graphs do not suggest substantial levels of pre-trending prior to SBHC adoption and lend support to our study design. All event-time bins from -5 to 5 are balanced on county. 6 is not.

eFigure 3. Two-Stage Event Study Estimates

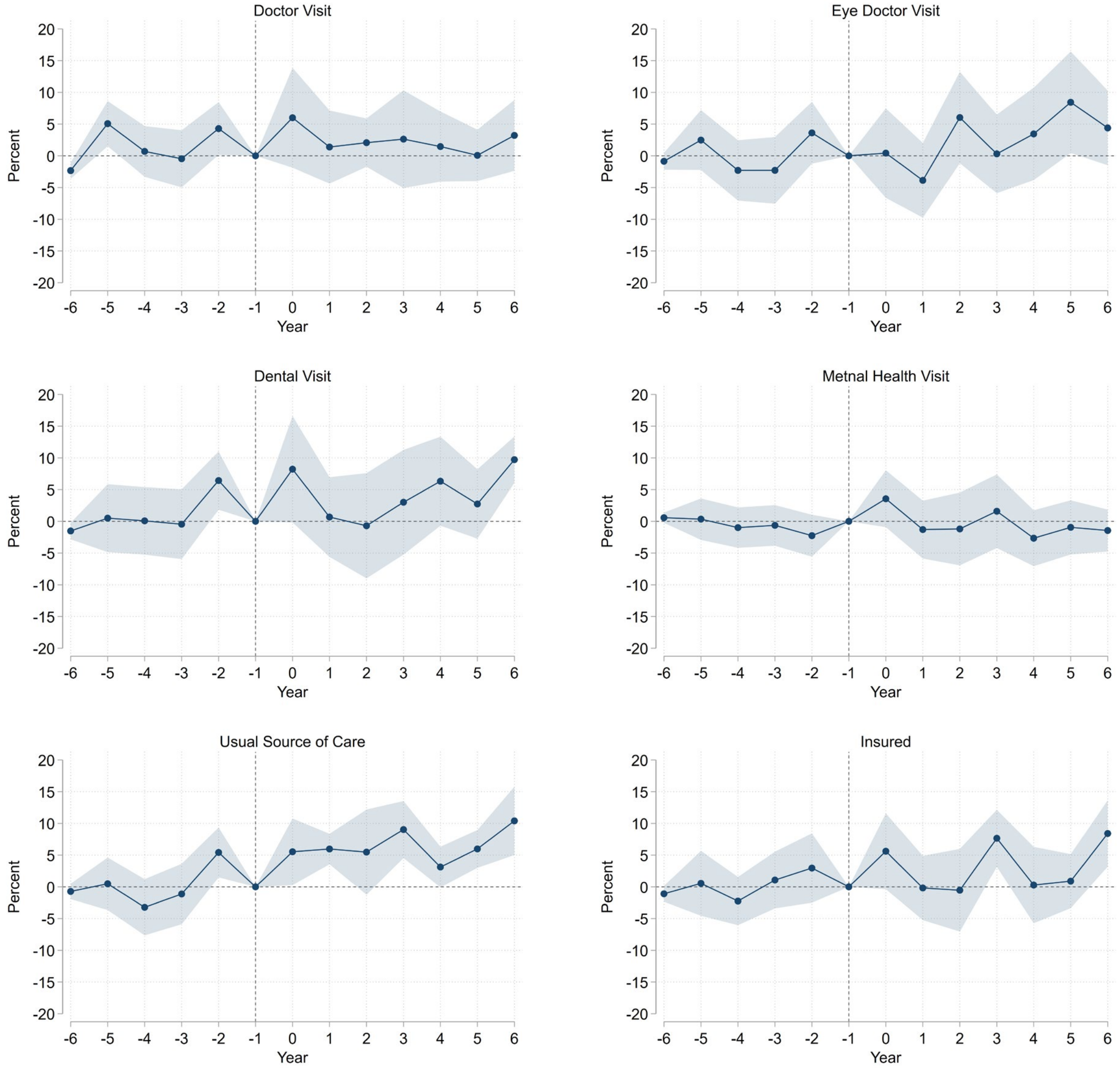

© 2023 Boudreaux M et al. *JAMA Network Open.*

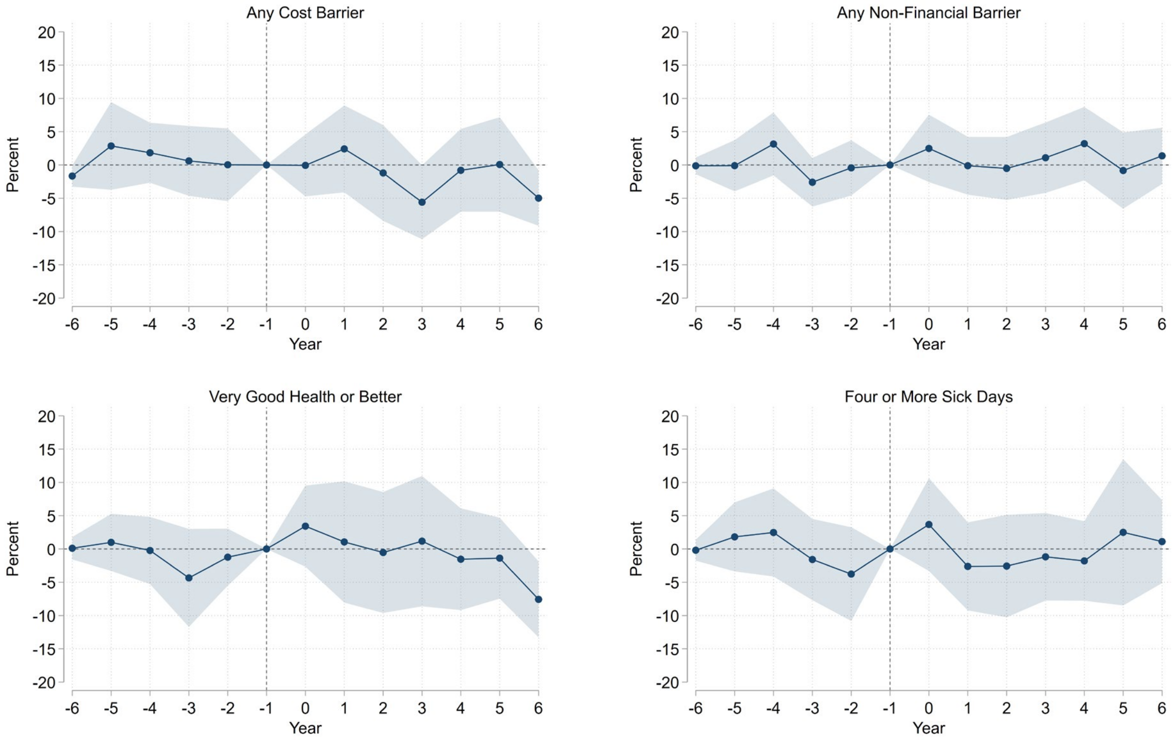

Source: 1997-2018 National Health Interview Survey merged with SBHC adoption indicators from the National Census for School Based Health. Estimates come from two-stage event study regressions.

© 2023 Boudreaux M et al. *JAMA Network Open.*

The coefficients plotted in eFigure 4 come from alternative specifications of our main difference-in-difference analysis in the low-income sample. These models make reasonable alternations to our main approach including adding state-by-year controls for childhood Medicaid income eligibility, removing all covariates, relaxing the balanced county requirement, removing the sample weights, and using standard OLS with two-way fixed effects. Our main approach also excluded counties that adopted prior to 2003. In addition, while that exclusion improves internal validity it may decrease external validity as the pre-2003 adoption counties had larger populations and slightly lower poverty rates than counties adopting in 2003-2013. We investigated if our results were sensitivity to the adoption window by expanding it to include counties that adopted in 2000-2017. We come to similar conclusions across alternatives.

eFigure 4. Sensitivity Analysis, Low-Income Children

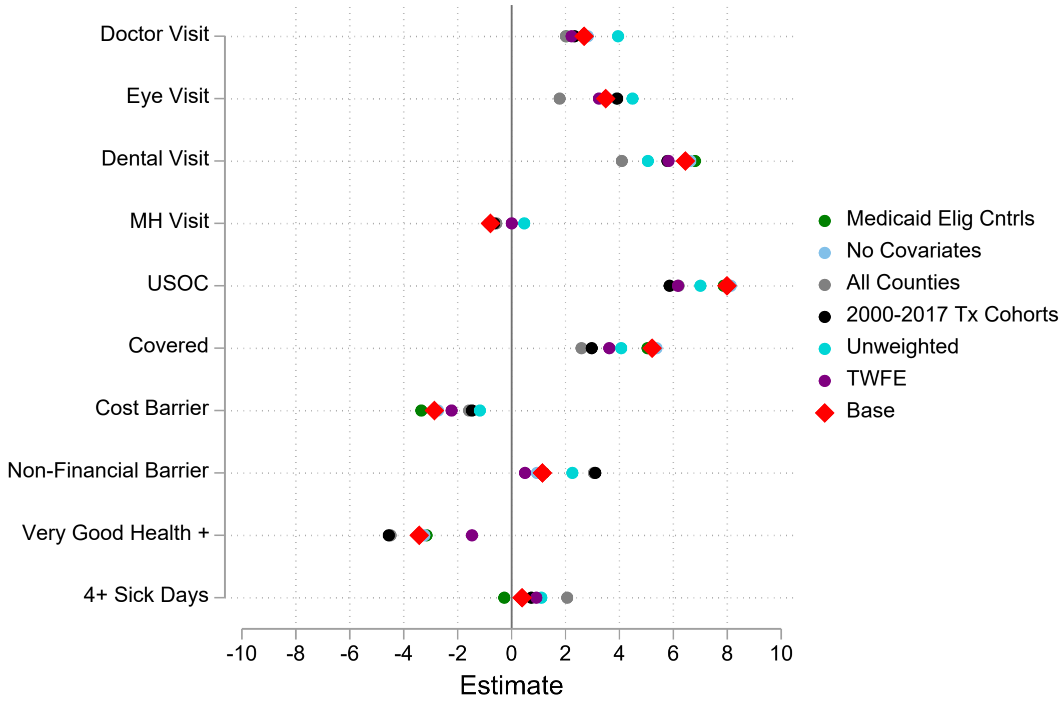

Source: 1997-2018 National Health Interview Survey merged with SBHC adoption indicators from the National Census for School-Based Health Centers.

© 2023 Boudreaux M et al. *JAMA Network Open.*

eTable 5 describes results from a two-way fixed effects model estimated using logistic regression. We report only results from the low-income sample. To our knowledge, the two-stage difference-in-differences procedure used in the main text has not been described for logistic regression. The results in eTable 2 are consistent with the conclusions we draw in the main manuscript.

**eTable 5. Two-way Fixed effects, Logistic Regression, Low-income Children age 5-17 years**		
	OR	95% CI
**Access and Use**		
Doctor/Health Professional Visit	1.1	(0.9-1.4)
Eye Doctor Visit	1.2	(0.9-1.6)
Dentist Visit	1.4	(1.1-1.6)
Mental Health Visit	1.0	(0.7-1.5)
Usual Source of Care	2.0	(1.5-2.7)
Insured	1.3	(0.9-1.1)
Any Financial Barrier	0.8	(0.6-1.1)
Any Non-Financial Barrier	1.0	(0.8-1.4)
**Health**		
Excellent/Very Good Health	0.9	(0.7-1.1)
4+ Missed School Days	1.1	(0.9-1.3)
Source: 1997-2018 National Health Interview Survey. OR is odds ratio. 95% CI is 95% confidence intervals.		

eTable 6 displays detailed results from the analysis examining the association between SBHCs and income-based disparities in usual source of care, dental visits, and insured status. Low-income is defined as under 200% FPL. The analysis was conducted by interacting the SBHC indicator with the low-income indicator in the full sample, in addition to county fixed effects, year fixed effects, and county*income and year*income effects. The later two terms allow each income group to have their own county and year effects. The table reports results from OLS and from the two-stage procedure described in the text. This was done because in the two-stage approach we are unable to recover the coefficient on low-income due it's presence in the first stage. However, both methods suggest that SBHCs are associated with reductions in disparities of a similar magnitude. The OLS coefficients in eTable 6 were used to produce Figure 3 in the main paper. Income-based disparities in the absence of SBHCs come from the coefficient on the low-income indicator and disparities with SBHCs are calculated as the sum of coefficients on low-income and the interaction of low-income and SBHC adoption. The relative reduction reported in the main text takes the form (Disparities without SBHC – Disparity with SBHC)/Disparity without SBHC*100.

eTable 6. The Association of SBHCs and Income-Based Disparities

	Usual Source of Care			Dental Visit			Insured		
	Est.	95% CI	P-Value	Est.	95% CI	P-Value	Est.	95% CI	P-Value
**TWFE**									
Low-Income	-7.4	(-19.3, 4.6)	0.226	-6.3	(-24.8, 12.2)	0.503	-5.5	(-19.9, 8.8)	0.45
Adoption County	-0.8	(-2, 0.3)	0.133	1.2	(-0.5, 2.9)	0.169	0.3	(-1, 1.7)	0.638
Low-Income*Adoption	7.2	(5.4, 9.1)	<0.001	4.9	(2, 7.7)	0.001	3.5	(1.3, 5.7)	0.002
**Two-Stage DD**									
Adoption County	-1.0	(-2.1, 0.2)	0.101	1.3	(-0.9, 3.5)	0.251	0.6	(-0.8, 2)	0.377
Low-Income*Adoption	8.9	(5.2, 12.7)	<0.001	5.2	(2.1, 8.3)	0.001	4.6	(0.3, 8.9)	0.036

Source: 1997-2018 National Health Interview Survey merged with SBHC adoption indicators from the National Census for School-Based Health Centers. Low-income is defined as under 200% of FPL. Models control for county fixed effect, year fixed effects, and county*income and year*income fixed effects.

© 2023 Boudreaux M et al. *JAMA Network Open.*
