# Supplementary material for: School-Based Health Centers, Access to Care, and Income-Based Disparities
Source: JAMA Netw Open. 2023 Sep 18;6(9):e2334532. doi: 10.1001/jamanetworkopen.2023.34532 (PMC10507491; doi:10.1001/jamanetworkopen.2023.34532)
Supplement: Supplement 2. — Data Sharing Statement [file jamanetwopen-e2334532-s002.pdf]

Data Sharing Statement

Boudreaux. School-Based Health Centers, Access to Care, and Income-Based Disparities. *JAMA Netw Open*. Published September 18, 2023. doi:10.1001/jamanetworkopen.2023.34532

Data
Data available: No

Additional Information
Explanation for why data not available: Data are based on restricted use files.
